# Functional analysis of human olfactory receptors with a high basal activity using LNCaP cell line

**DOI:** 10.1371/journal.pone.0267356

**Published:** 2022-04-21

**Authors:** Takashi Ieki, Yuki Yamanaka, Keiichi Yoshikawa

**Affiliations:** Sensory Science Research, Kao Corporation, Haga, Tochigi, Japan; Monell Chemical Senses Center, UNITED STATES

## Abstract

Humans use a family of more than 400 olfactory receptors (ORs) to detect odorants. However, deorphanization of ORs is a critical issue because the functional properties of more than 80% of ORs remain unknown, thus, hampering our understanding of the relationship between receptor function and perception. HEK293 cells are the most commonly used heterologous expression system to determine the function of a given OR; however, they cannot functionally express a majority of ORs probably due to a lack of factor(s) required in cells in which ORs function endogenously. Interestingly, ORs have been known to be expressed in a variety of cells outside the nose and play critical physiological roles. These findings prompted us to test the capacity of cells to functionally express a specific repertoire of ORs. In this study, we selected three cell lines that endogenously express functional ORs. We demonstrated that human prostate carcinoma (LNCaP) cell lines successfully identified novel ligands for ORs that were not recognized when expressed in HEK293 cells. Further experiments suggested that the LNCaP cell line was effective for functional expression of ORs, especially with a high basal activity, which impeded the sensitive detection of ligand-mediated activity of ORs. This report provides an efficient functional assay system for a specific repertoire of ORs that cannot be characterized in current cell systems.

## Introduction

Olfaction, the sense of smell, plays an important role in recognizing odors and inspiring preferences for products. The discovery of olfactory receptors (ORs) in olfactory sensory neurons (OSNs) has initiated the molecular era of olfactory research [1]. Humans use a family of approximately 400 ORs, each of which recognizes a unique subset of odorants with distinct affinities [2]. To date, 12% of human ORs have been functionally characterized and matched with their cognate ligands [3–8]. This progress has not only furthered the understanding of olfactory coding but also provided opportunities for industrial applications. The number of patents on ORs has drastically increased over the past three decades. However, recent advances in understanding olfaction and technological development seem to have reached a plateau, probably due to a large number of uncharacterized ORs.

The understanding of the functions of OR has progressed slowly, and a well-recognized reason is the difficulty in expressing ORs on the surface of heterologous cells. Most exogenous OR proteins are retained in the endoplasmic reticulum, following which they are degraded [9]. Several approaches have been successfully established to promote cell surface expression of ORs in heterologous cell systems. Fusing an N-terminal epitope tag from bovine rhodopsin improves the membrane trafficking of OR proteins [10]. Co-expression of chaperones, such as receptor transporting protein 1 (RTP1) and other G protein-coupled receptors (GPCRs), in OSNs promotes membrane trafficking of ORs in heterologous cells [10–13]. Signal amplifiers are also effective in detecting weak levels of signal transduction derived from a small number of cell surface ORs [14, 15]. Although these approaches resulted in the deorphanization of a significant number of ORs, more than 80% of ORs are still orphan [7], indicating that heterologous cells lack additional factor(s) required for the functional expression of ORs.

A promising approach to overcome this difficulty is the use of homologous systems that naturally equip the cell with factors required for cell surface expression and signal transduction of the entire repertoire of ORs [16, 17]. However, the success achieved through this approach has been limited, especially for human ORs. Genetic manipulation of animals, in which defined OR-expressing neurons are labeled with fluorescent probes or in which an activity-dependent fluorescent reporter is expressed in OSNs, has identified pairs of ORs and their ligands [18–22]. However, these approaches are low throughput, and they cannot be easily applied to the characterization of many human ORs. Application of recent approaches for the *in vivo* identification of OR repertoires has been restricted to mouse ORs [23, 24]. An immortalized cell line derived from the OSN lineage appears to be a powerful tool to functionally express a given OR; however, it has not been widely utilized probably due to genetic instability and difficulty in preventing differentiation and maintaining the molecular characteristics of normal OSN [25].

Interestingly, evidence suggests that the expression of ORs is not only restricted to OSNs but is also apparent in non-olfactory tissues. This expression has been observed in a variety of tissues, such as the heart, lung, sperm, skin, and cancerous tissues, and cell lines [26–36]. The repertoire of non-nasally expressed ORs appears to vary between tissues [27, 29]. At least a part of the non-nasal ORs have been proven to be functional and play a physiological role, indicating that the non-nasal cells have a molecular machinery that sufficiently promotes the functional expression of a distinct repertoire of ORs [31–37]. Therefore, we conducted this study to test a series of potential cell lines that can endogenously and functionally express ORs that can be used as a novel functional expression system for human ORs.

## Materials and methods

### Expression vector

Human ORs and other genes were cloned as described earlier [38, 39]. Briefly, all human OR genes and trace amine-associated receptor (TAAR) genes were amplified from human genomic DNA (Promega, Madison, WI, USA). The identified single nucleotide polymorphisms (SNPs) that were different from the reference sequences in GenBank (http://www.ncbi.nlm.nih.gov/genbank/) or HORDE (http://genome.weizmann.ac.il/horde/) but were found in the NCBI dbSNP database (http://www.ncbi.nlm.nih.gov/SNP/) were not modified (S1 Table). When we amplified an OR gene with unknown missense mutations, we modified them to reference sequences. Each of the amplified receptor genes was inserted into the pME18S vector to generate OR proteins fused with N-terminal epitope tags of FLAG and 20 N-terminal amino acids of bovine rhodopsin. For the primary screening, which was performed on 378 of the 412 ORs, we screened HEK293 cells against each of the 6 odorants (S2 Table), and the remaining 34 ORs were tested during later screening (S3 Table) via construction of expression vectors. The 34 receptors were 1F12, 2AP1, 2T35, 2V1, 2Y1, 4K3, 4K5, 4Q2, 5AC1, 5AL1, 5AV1, 5G3, 6P1, 8S1, 10D3, 10J4, 11H2, 11H7, 14A16, 14C36, 14I1, 14J1, 52E1, 52E5, 52N4, 52Z1, 56A5, TAAR1, TAAR2, TAAR3, TAAR5, TAAR6, TAAR8, and TAAR9. The human RTP1S gene was amplified from human genomic DNA (Promega, Madison, WI, USA), and inserted into pME18S without any N-terminal epitope tag. The human phosphodiesterase 1C (PDE1C, NM_001191059) gene was amplified from cDNA derived from the human lung (BioChain Institute, Newark, CA, USA). cDNA of M3AchR was purchased from Thermo Fisher Scientific (Waltham, MA, USA), and the ORF was inserted into pME18S containing both the N-terminal epitope tags of FLAG and 20 amino acids of bovine rhodopsin.

### Cells

HEK293 cells were provided by Prof. Touhara Kazushige at the University of Tokyo; HepG2, HuH7, and LNCaP cells were purchased from ECACC, JCRB cell bank (JCRB0403), and ATCC (LNCaP clone FGC, ATCC CRL-1740), respectively. HEK293 and HepG2 cells were grown in Dulbecco’s modified Eagle’s medium (DMEM, 4.5 g/L glucose, Nacalai Tesque, Kyoto, Japan) supplemented with 10% fetal bovine serum (FBS). Antibiotic-antimycotic (1%, 100X, Thermo Fisher Scientific, Waltham, MA, USA) was applied for HepG2 cells. HuH7 cells were grown in DMEM (low glucose, pyruvate, Thermo Fisher Scientific. Waltham, MA, USA) supplemented with 10% FBS, and LNCaP cells were grown in RPMI1640 medium (ATCC modification, Thermo Fisher Scientific, Waltham, MA, USA) supplemented with 10% FBS. All cells were cultured at 37°C in a humidified atmosphere containing 5% CO_2_. Cell cultures were split using 0.25% trypsin-EDTA (Invitrogen, Waltham, MA, USA) every two to six days before reaching confluence.

### cAMP response element (CRE)-regulated luciferase reporter gene assay

Transfection and luciferase assays were performed as previously described with some modifications [38, 39]. For the 96-well plate assay for identifying ORs for six odorants in HEK293 cells, 0.075 μg of a FLAG-Rho-tagged OR pME18S vector, 0.03 μg of CRE/luc2PpGL4.29 (CRE-dependent firefly luciferase), 0.03 μg of pRL-CMV (constitutively expressed Renilla luciferase), and 0.03 μg of RTP1S pME18S vector per well were transfected in HEK293 cells. To compare between HEK293 cells and other three cell lines, 0.075 μg of a FLAG-Rho-tagged OR pME18S vector, 0.015 μg of CRE/luc2PpGL4.29, 0.015 μg of pRL-CMV, 0.03 μg of RTP1S pME18S, and 0.03 μg of Gαolf pME18S vector per well were transfected into each cell line with the transfection reagent in poly d-lysine-coated 96-well plates (Corning, NY, USA). When co-transfecting with PDE1C, the volume of pRL-CMV was reduced by the same amount, to maintain a constant total volume of the transfected plasmid. The transfection reagent differed depending on the cell type. For HEK293 cells, 0.41 μL/well of 0.1% polyethylenimine Max (PEI-MAX, Polysciences, Warrington, PA, USA) was applied. PEI-MAX was diluted with distilled water, and its pH was adjusted to 7.4. Further, 0.2 μL/well and 0.35 μL/well of Lipofectamine 2000 (Thermo Fisher Scientific, Waltham, MA, USA) were used for HuH7 and HepG2 cells, respectively, and 0.15 μL/well of Lipofectamine 3000 (Thermo Fisher Scientific, Waltham, MA, USA) was used for LNCaP cells. We added 2 μL of P3000 reagent per 1 μg plasmid when we used Lipofectamine 3000. As a buffer for transfection reagents and plasmids, DMEM was used for HEK293 and HuH7 cells, and Opti-MEM was used for HepG2 and LNCaP cells. After incubation for 20 min, 90 μL of the cell suspension (2 × 10^5^ cells/cm^2^ in each growth medium) was added to 10 μL of the above transfection solution per well, and the plate was incubated at 37°C in 5% CO_2_ for 24 h.

For the 384-well plate assay, 0.029 μg of a FLAG-Rho-tagged OR pME18S vector, 0.022 μg of CRE/luc2PpGL4.29, 0.0011 μg of pRL-CMV, and 0.012 μg of RTP1S pME18S vectors per well were transfected into HEK293 cells with the transfection reagent in poly D lysine-coated 384-well black plates (Corning). Following this, 0.029 μg of a FLAG-Rho-tagged OR pME18S, 0.011 μg of CRE/luc2PpGL4.29, 0.0011 μg of pRL-CMV, 0.012 μg of RTP1S pME18S, and 0.010 μg of Gαolf pME18S vectors per well were transfected into the other three cell lines. The presence or absence of Gαolf was determined based on our previous results that Gαolf did not improve detection of OR-mediated cAMP response in HEK293 cells but did so in other types of cells [15, 40–42]. Thus, cell lines other than HEK293 cells were tested with Gαolf. Regarding transfection reagents, 0.16 μL/well of PEI-MAX (0.1%, pH 7.5) was added to HEK293 cells, 0.14 μL/well or 0.077 μL/well of Lipofectamine 2000 was applied to HepG2 or HuH7 cells, respectively, and Lipofectamine 3000 (0.058 μL/well) was added to LNCaP cells. Identical buffers, as described above, were used to dilute the transfection reagents and plasmids. After incubation for 20 min, 40 μL of cell suspension (2 × 10^5^ cells/cm^2^ in each growth medium) was added to 4.4 μL of the above transfection solution per well, and the solution was incubated at 37°C in 5% CO_2_ for 24 h. The 384-well plate assay was performed using the BiomekFX laboratory automation system (Beckman Coulter, Brea, CA, USA).

After 24 h of transfection, the medium was removed, and the transfected cells were treated with an odorant solution diluted in the growth medium without FBS. The odorant solution diluted in Ringer’s solution (140 mM NaCl, 5 mM KCl, 1 mM MgCl_2,_ 2 mM CaCl_2,_ 10 mM HEPES, 5 mM glucose, pH 7.4) was also used for HEK293 cells to reduce the background signal from transfected ORs. The 96- or 384-well plates were sealed, and they were incubated at 37°C for 4 h. The luciferase reporter gene activities were measured using a Dual-Glo Luciferase Assay System (Promega, Madison, USA), and luminescence was measured with Mithras LB940 (Berthold Technologies, Bad Wildbad, Germany) for the 96-well plate and with Ensight multimode plate reader (PerkinElmer, Waltham, MA, USA) for the 384-well plate.

### Odorants

*l*-Carvone, anis aldehyde, methyl β-naphthyl ketone, *d*-carvone, and linalool were purchased from FUJIFILM Wako Pure Chemicals (Osaka, Japan), and *cis*-3-hexenol was purchased from Merck (Darmstadt, Germany). These six odorants were used in both single and mixed solutions. For primary screening of ORs, the tested concentrations were as follows: *l*-carvone (300 μM), *cis*-3-hexenol (1 mM), anis aldehyde (3 mM), methyl β-naphthyl ketone (100 μM), *d*-carvone (300 μM), and linalool (3 mM). The mixture comprised 100 mM *l*-carvone, *cis*-3-hexenol, methyl β-naphthyl ketone, *d*-carvone, 300 mM anis aldehyde, and linalool in EtOH, and it was diluted with the assay medium for stimulation. The final concentrations of odorant mixture applied to OR screening were as follows: *l*-carvone (100 μM), *cis*-3-hexenol (100 μM), anis aldehyde (100 μM), methyl β-naphthyl ketone (100 μM), *d*-carvone (300 μM), and linalool (300 μM). For the dose-response analyses of odorants, a stock solution (1 M) of each compound was prepared in EtOH and subsequently diluted with the growth medium without FBS. Control stimulation without an odorant employed the highest concentration of EtOH used for each odorant (i.e., the solution for stimulation with 0 μM l-carvone contained 0.3% EtOH).

### Immunocytochemistry (live-cell staining)

A 35-mm dish (IWAKI, Shizuoka, Japan) was coated with 0.01% poly d-lysine PBS solution for 15 min. After incubation, the dish was washed with distilled water thrice and then used. 1.5 μg of a FLAG-Rho-tagged OR pME18S vector and 0.6 μg of RTP1S pME18S plasmids were transfected. Following this, 8.4 μL of 0.1% PEI-MAX solution was added to HEK293 cells, and 3.04 μL of Lipofectamine 3000 and 2 μL of P3000 reagent per 1 μg plasmid were used for LNCaP cells. The plasmids and transfection solutions were mixed in 200 μL Opti-MEM (Invitrogen, Waltham, MA, USA), and incubated for 20 min. After incubation, 1 mL of cell suspension (2 × 10^5^ cells/cm^2^ in each growth medium) was added to 200 μL of the aforementioned transfection solution per dish, and the solution was incubated at 37°C in 5% CO_2_ for 24 h.

After 24 h of transfection, the dish was placed on ice until PBS was added. First, the medium was removed, and the dish was incubated with a mouse anti-FLAG antibody (1:1000, KO602-S, Trans genic Inc. Fukuoka, Japan) for 1 h. The medium used for HEK293 cells was DMEM with 10% FBS and 10 mM HEPES (pH 7.5) and that for LNCaP cells was RPMI1640 with 10% FBS. After washing with Ringer’s solution thrice, the dish was incubated with a goat anti-mouse IgG-Alexa Fluor 488 antibody (1:500, Invitrogen, Waltham, MA, USA) for 1 h. The dish was then washed again with Ringer’s solution thrice. After washing, the cells in the dish were fixed with 1% PFA/PB for 15 min. Finally, the dish was washed with PBS three times, and PBS was allowed to remain in the dish. Fluorescent images were obtained using a microscope (BZ-X700, Keyence, Osaka, Japan).

### Data analysis

Data analysis was performed using Microsoft Excel or GraphPad Prism software. Fold increase was calculated as Luc(S) divided by Luc(NS), where Luc(S) was the luminescence intensity of firefly luciferase divided by the luminescence intensity of Renilla luciferase of a certain odorant-stimulated well and Luc(NS) was the luminescence intensity of firefly luciferase divided by the luminescence intensity of Renilla luciferase of a certain non-stimulated well. We excluded the data with higher concentrations of odorants which induced cell toxicity, defined as statistically significant reduction of the luminescence intensity of Renilla luciferase compared with that of non-stimulated control cells (*P*<0.05, Student’s *t*-test). Data were fitted to a sigmoidal curve using the GraphPad Prism software.

## Results

**Fig 1:**
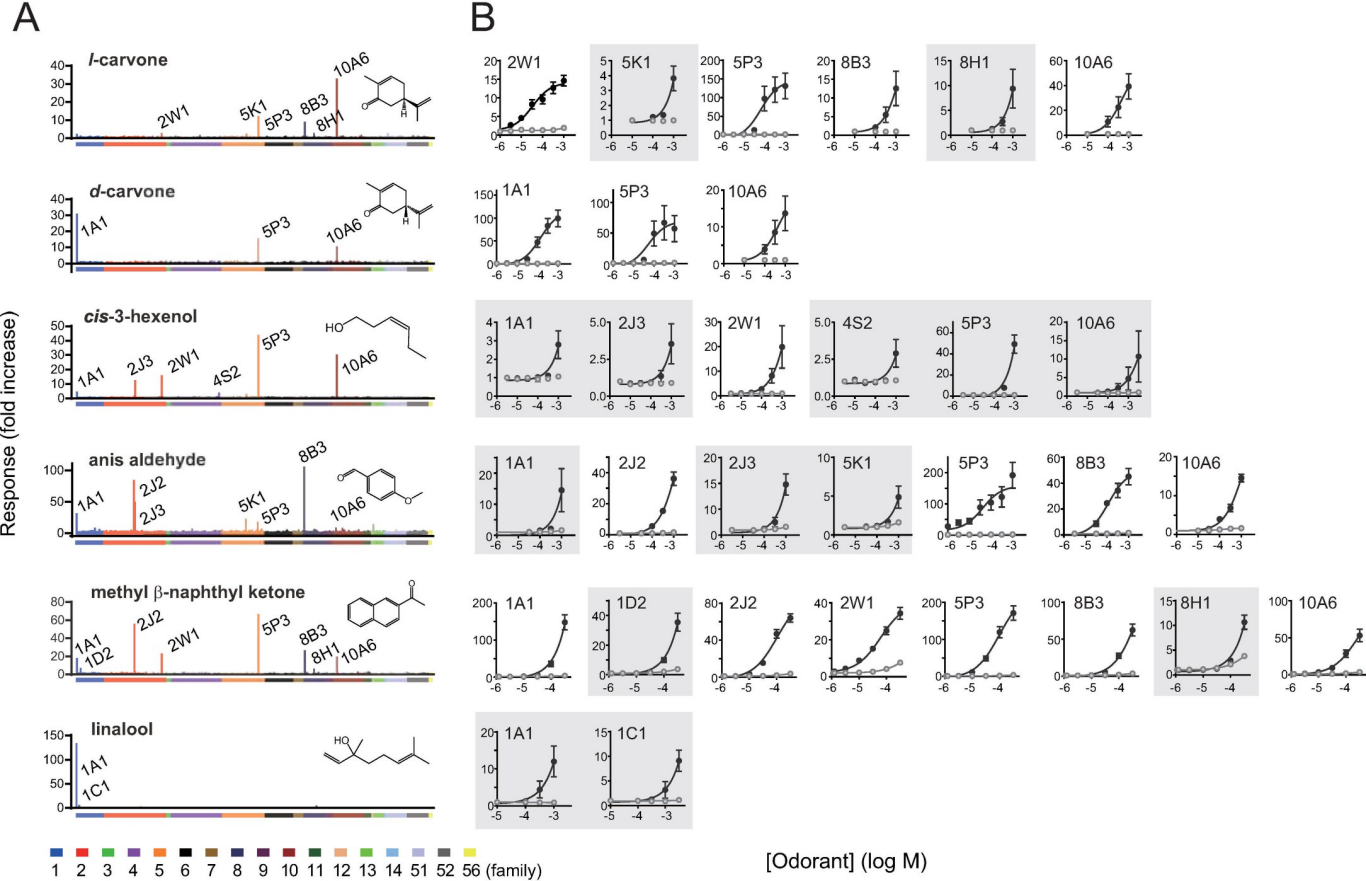
HEK293 cell-based screening of human ORs for six perfumery raw materials. A. Primary screening of human ORs using 384-well plates. 378 ORs were expressed in HEK293 cells, and they were stimulated with a single concentration of each odorant. HEK293 cells were co-transfected with each OR, CRE/luc2PpGL4.29, pRL-CMV, and RTP1S. Odorant solutions were diluted in the growth medium without FBS. X-axis lists each of the 378 ORs, and color bars represent OR families. Y-axis indicates the response of cells expressing each OR as fold increase where a signal from stimulated cells was divided by that from non-stimulated cells expressing the same OR (mean values from two screening replicates). B. Verification of dose-dependent responsiveness of cell-expressing candidate ORs using 96-well plates. Each OR was co-transfected with CRE/luc2PpGL4.29, pRL-CMV, and RTP1S. 32 pairs of ORs and their agonists that meet our statistical criteria for dose-dependent responses are shown. The grey-shaded ORs do not fit a sigmoidal curve because their responses were not saturated in the range of concentrations tested due to less sensitivity. Y-axis indicates the fold increase value, and data are shown as mean ± SE from three independent experiments.

Six odorants commonly used as perfumery raw materials, namely *l*-carvone for mint, *d*-carvone for caraway seeds, *cis*-3-hexenol for green, anis aldehyde for spicy/anisic, methyl β-naphthyl ketone for rose, and linalool for citrus, were selected. First, we used HEK293 cells, the most common heterologous cell system, to investigate ORs that were sensitive to odorants [3–6, 43]. As an assay platform for ORs, we selected a CRE-regulated luciferase reporter gene assay, which is the most commonly used for detecting cAMP signals mediated by activated ORs in HEK293 cells [3, 4, 6, 38, 39, 44]. For the primary screening, HEK293 cells were transfected with 378 ORs and stimulated with a single concentration of each of the six odorants (Fig 1A and S2 Table). The response of cells expressing each OR was presented as fold increase, where a signal from stimulated cells was divided by that from non-stimulated cells expressing the same OR. We then selected ORs that showed response amplitudes (fold increase) above the mean+SD among all the examined ORs in the primary screening and conducted follow-up dose-response analysis (Fig 1B). We concluded that an OR is a receptor for the odorant if at least one concentration of the odorant produced a statistically significant response in OR-expressing cells as compared to both the OR-expressing cells stimulated with medium alone and vector-transfected control cells stimulated with the concentration of the odorant (Sidak–Bonferroni method with alpha = 5.0%). The identified ORs were as follows: OR2W1, OR5K1, OR5P3, OR8B3, OR8H1, and OR10A6 for *l*-carvone; OR1A1, OR5P3, and OR10A6 for *d*-carvone; OR1A1, OR2J3, OR2W1, OR4S2, OR5P3, and OR10A6 for *cis*-3-hexenol; OR1A1, OR2J2, OR2J3, OR5K1 OR5P3, OR8B3, and OR10A6 for anis aldehyde; OR1A1, OR1D2, OR2J2, OR2W1, OR5P3, OR8B3, OR8H1, and OR10A6 for methyl β-naphthyl ketone; and OR1A1 and OR1C1 for linalool. Negative ORs that were tested in this follow-up dose-response analysis but did not show reproducible responses were as follows: OR51A7 for *l*-carvone and *d*-carvone; OR2J2 for *cis*-3-hexenol; OR13C2, OR10G4, and OR1L6 for anis aldehyde; and OR11H6, OR10Q1, and OR2S2 for methyl β-naphthyl ketone (S2 Fig). These data not only provided 25 novel pairs of ORs and their ligands (white circles in Table 1), contributing to future studies to understand the relationship between receptor function and perception, but also provide a basis for evaluating the capability of novel heterologous cell systems other than HEK293 cells.

**Table 1:** ORs for each odorant^a^.

	1A1	1C1	1D2	1N2	2J1	2J2	2J3	2W1	4S2	5K1	5P3	6P1	8B3	8H1	10A6	14J1	51T1
l-carvone	▲^4,38)^							●^3,4)^		O	●		●^3)^	O	O		O
*d-*carvone	●^3,4)^							●^4)^			O		▲^3)^		O		O
cis-3-hexenol	O				▲^3)^	▲^3)^	●^3)^	●^3)^	O		O				O	▲^3)^	O
anis aldehyde	O					O	O			O	O	▲^3)^	O		O		O
m-b-n-ketone	O		O			O	▲^38)^	O			O		O	O	O		O
linalool	O	●^3)^		▲^3)^													O

^a^m-β-n-ketone, methyl β-naphthyl ketone; O, Newly identified in this study; ▲, Previously identified but not detected in this study; ●, Previously identified and consistently detected in this study; Number in parenthesis, reference

We then examined whether the three cell lines, HepG2 (human hepatocellular carcinoma cells), HuH7 (human hepatocellular carcinoma cells), and LNCaP (human prostate carcinoma cells), all of which express functional ORs, were effective as an alternative expression system in comparison with HEK293 cells. OR1A1 is expressed in HepG2 cells, and it responds to *l*-carvone [45]. OR1A2 is expressed in HuH7 cells, and it is sensitive to (-)-citronellal and citronellol [46]. LNCaP cells express OR51E1 and OR51E2 (also named prostate-specific G-protein-coupled receptor [PSGR]), which recognize β-ionone, propionic acid, butyric acid, and nonanoic acid [47, 48]. As HepG2, HuH7, and LNCaP have not been widely utilized as functional expression systems for GPCRs, we first screened several experimental conditions, such as transfection reagents and culture buffers, based on the responsiveness of OR1A1 (S1 Fig).

**Fig 2:**
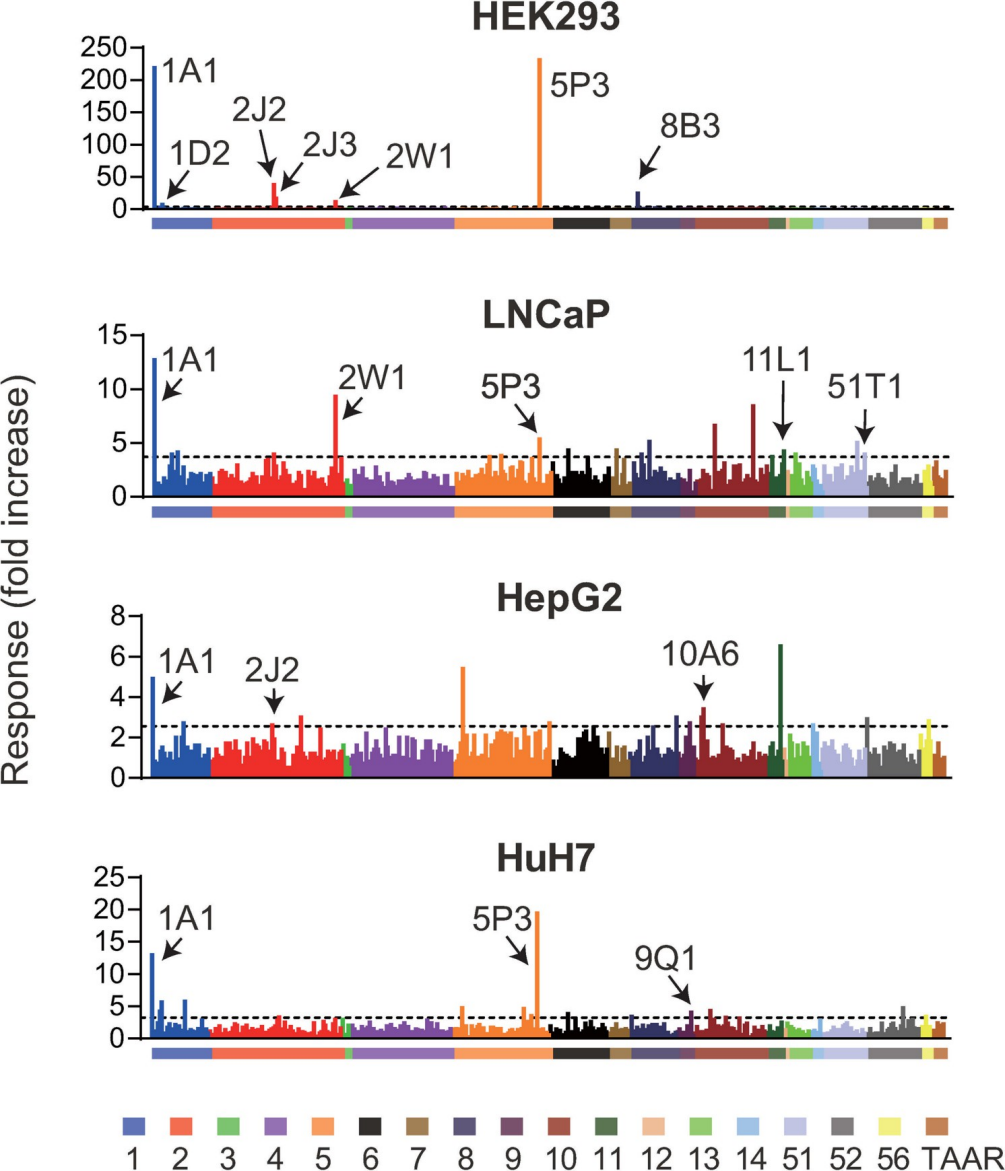
Capacity of three cell lines as a functional expression cell system for ORs. A total of 412 ORs were expressed in different cell lines. Each OR was co-transfected with CRE/luc2PpGL4.29, pRL-CMV, and RTP1S in HEK293 cells whereas Gαolf was also co-transfected in the other three cell lines. They were stimulated with a mixture of six odorants in the growth medium without FBS. The X-axis lists each of the 412 ORs, and color bars represent OR families. Y-axis indicates the response of cells expressing each OR as a fold increase. Data are presented as the mean values from two screening replicates. The dashed line denotes mean + 2SD of all the examined ORs except for those validated in HEK293 cells. The name of the receptor is described if the response value in the follow-up dose-response analysis meets our criteria (S3–S6 Figs).

Under optimized conditions, we tested whether the three cell lines could functionally express a unique subset of ORs, including those that could not be expressed in HEK293 cells. The cell lines were transfected with 412 ORs, including TAARs (see Materials and Methods), and they were stimulated with a mixture of the 6 odorants. Measuring activation against the odorant mixture suggested that each cell line was capable of functionally expressing a distinct set of ORs (Fig 2 and S3 Table). We tentatively identified activated ORs based on the criterion that the response amplitude (fold increase) should be above mean + 2SD of all the examined ORs except for those validated in HEK293 cells (Fig 1, 12 ORs sensitive to each component of the mixture in HEK293 cells). Then we conducted follow-up experiments testing higher and lower concentrations of the odorant mixture (S3 Fig). As a result, ORs were identified as positive if at least one concentration of the odorant produced a statistically significant response of an OR-expressing cell as compared to that of cells stimulated with medium alone and a vector-transfected control cell (Sidak–Bonferroni method with alpha = 5.0%), labeled in Fig 2. We identified OR1A1, OR1D2, OR2J2, OR2J3, OR2W1, OR5P3, and OR8B3 as ORs sensitive to the mixture using HEK293 cells, which included 7 out of 12 ORs identified in Fig 1. Some of the ORs that were activated against each component of the mixture (Fig 1) were undetectable in this experiment likely due to the low concentration of the tested component in the mixture (see Materials and Methods) and/or antagonistic effects [31, 49–51]. The same experiments using three other cell lines resulted in the identification of OR1A1, OR2J2, and OR10A6 from HepG2; OR1A1, OR5P3, and 9Q1 from HuH7; and OR1A1, OR2W1, OR5P3, 11L1, and OR51T1 from LNCaP (Fig 2 and S4–S6 Figs). The response amplitudes and errors of the identified ORs in three cell lines were smaller than those in HEK293 cells, suggesting a difference in capacity of cAMP metabolism between cell lines. These data provide evidence of a unique pattern of ORs functionally expressed in each cell line. Notably, activation of OR9Q1 and OR51T1, which are orphan receptors, was specifically detected in HuH7 cells or LNCaP cells but not in HEK293 cells (Figs 1 and 2). This result suggests that these cell lines are candidate functional expression systems for ORs non-functional in HEK293 cells. Here, we focused on OR51T1 and LNCaP cell pairs with the most robust response among those of the orphan ORs.

**Fig 3:**
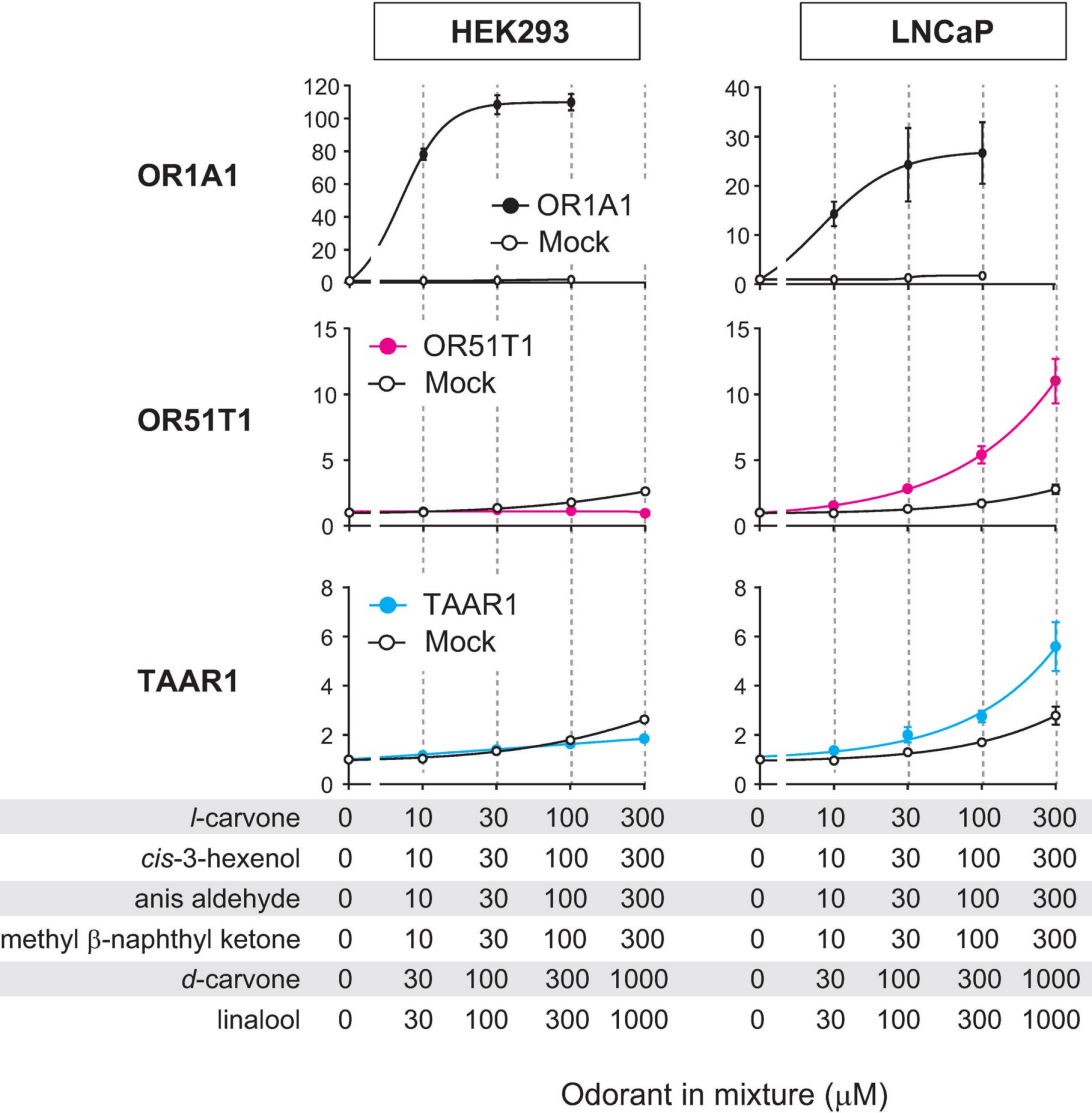
Activations of specific ORs were selectively monitored in LNCaP cells. Dose-response curves of OR1A1 (top, black), OR51T1 (middle, magenta), and TAAR1 (bottom, blue) expressed in HEK293 (left) or LNCaP cells (right) against increasing concentrations of the odorant mixture. Each OR was co-transfected with CRE/luc2PpGL4.29, pRL-CMV, RTP1S and Gαolf. X-axis indicates final concentrations of each of the six components of the mixture. Ringer’s solution was used to dissolve the odorant mixture. Assays were also conducted on mock-transfected cells transfected with empty vector, CRE/luc2PpGL4.29, pRL-CMV, RTP1S, and Gαolf. (white). Y-axis indicates the fold increase in response. Data are shown as mean ± SE of three independent experiments.

**Fig 4:**
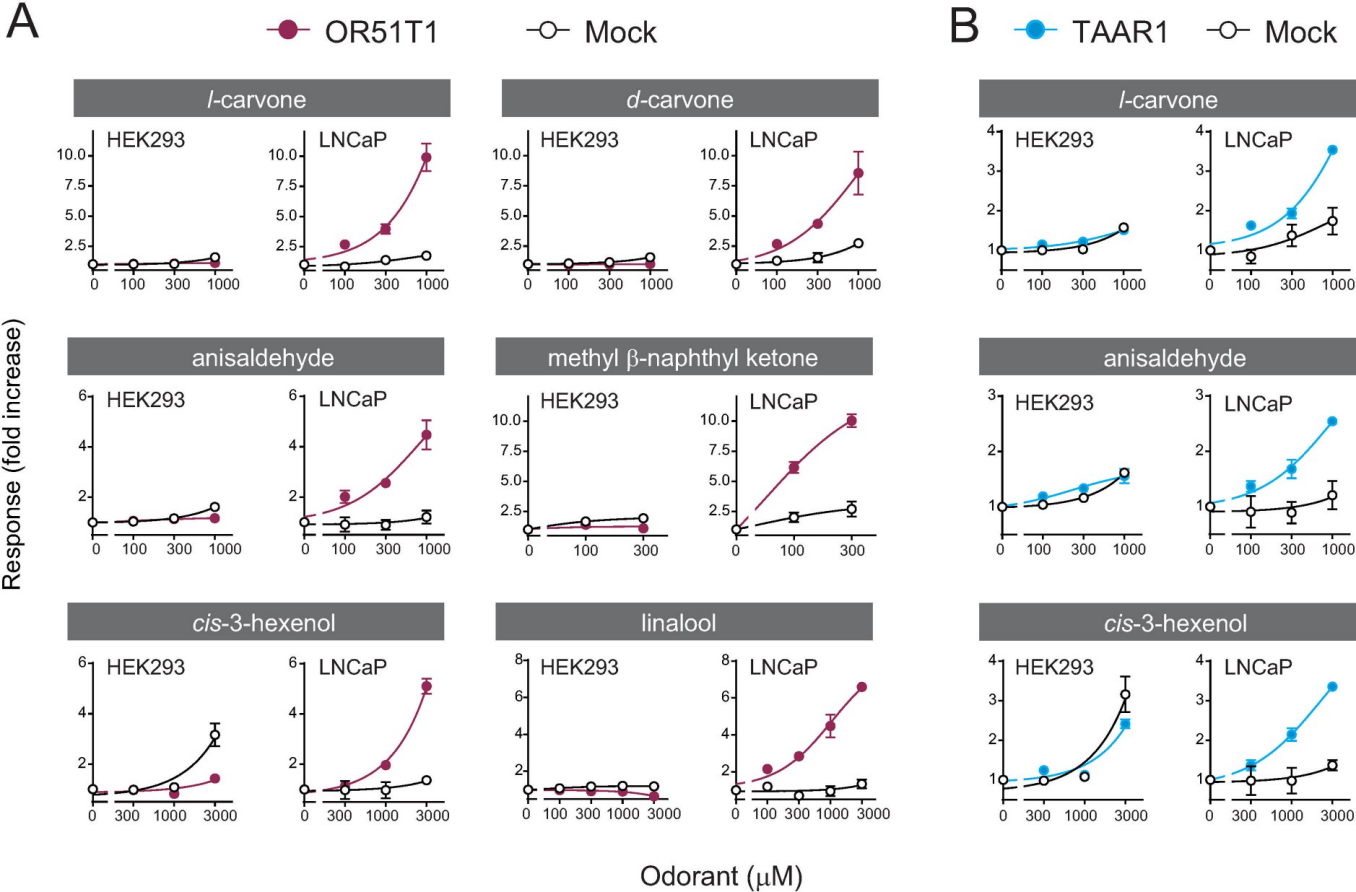
Identification of novel pairs of ligands and ORs in LNCaP cells. Dose-response curves of OR51T1 (A, magenta) and TAAR1 (B, blue) expressed in HEK293 (left) or LNCaP cells (right) against increasing concentrations of each odorant. Each OR in both cell lines was co-transfected with CRE/luc2PpGL4.29, pRL-CMV, RTP1S and Gαolf. Ringer’s solution was used to dissolve the odorant mixture. Y-axis indicates the fold increase in response. Data are shown as mean ± SE of three independent experiments.

When the odorant mixture was tested at high concentrations, OR51T1 activation was still not monitored in HEK293 cells; however, it was robustly detected in LNCaP cells (Fig 3). This result was in contrast with that for OR1A1, where HEK293 cells as well as LNCaP cells showed an OR1A1-mediated response in a dose-dependent manner. A potential reason for LNCaP-selective detection of OR51T1 activation was co-expression of Gαolf. We expressed Gαolf in three cell lines including LNCaP cells but did not in HEK293 cells because previous studies indicated that co-expression of Gαolf improved detection of OR activation from specific cell types, except for HEK293 cells [15, 40–42]. However, co-expression of Gαolf was not required for sensitive detection of OR activation in LNCaP cells, indicating that the observed difference in activation of ORs expressed in HEK293 cells and LNCaP cells is due to an inherent capacity of the cells rather than the presence or absence of exogenous Gαolf (S7 Fig). Following this, we tested six components of the odorant mixture. OR51T1-expressing LNCaP cells showed a significant dose-dependent response to all components of the mixture, leading to the identification of novel pairs of odorant-ORs that could not be achieved using HEK293 cells (Fig 4). No responses were observed in LNCaP cells without OR51T1 (mock-transfected cells), indicating that the observed responses were dependent on OR51T1 expression. OR51T1 was insensitive to odorants with quite different structures from the identified agonists (S8 Fig). This result demonstrates the deorphanization of OR51T1, and it supports the idea that LNCaP is an efficient cell line for functional expression of at least one OR.

**Fig 5:**
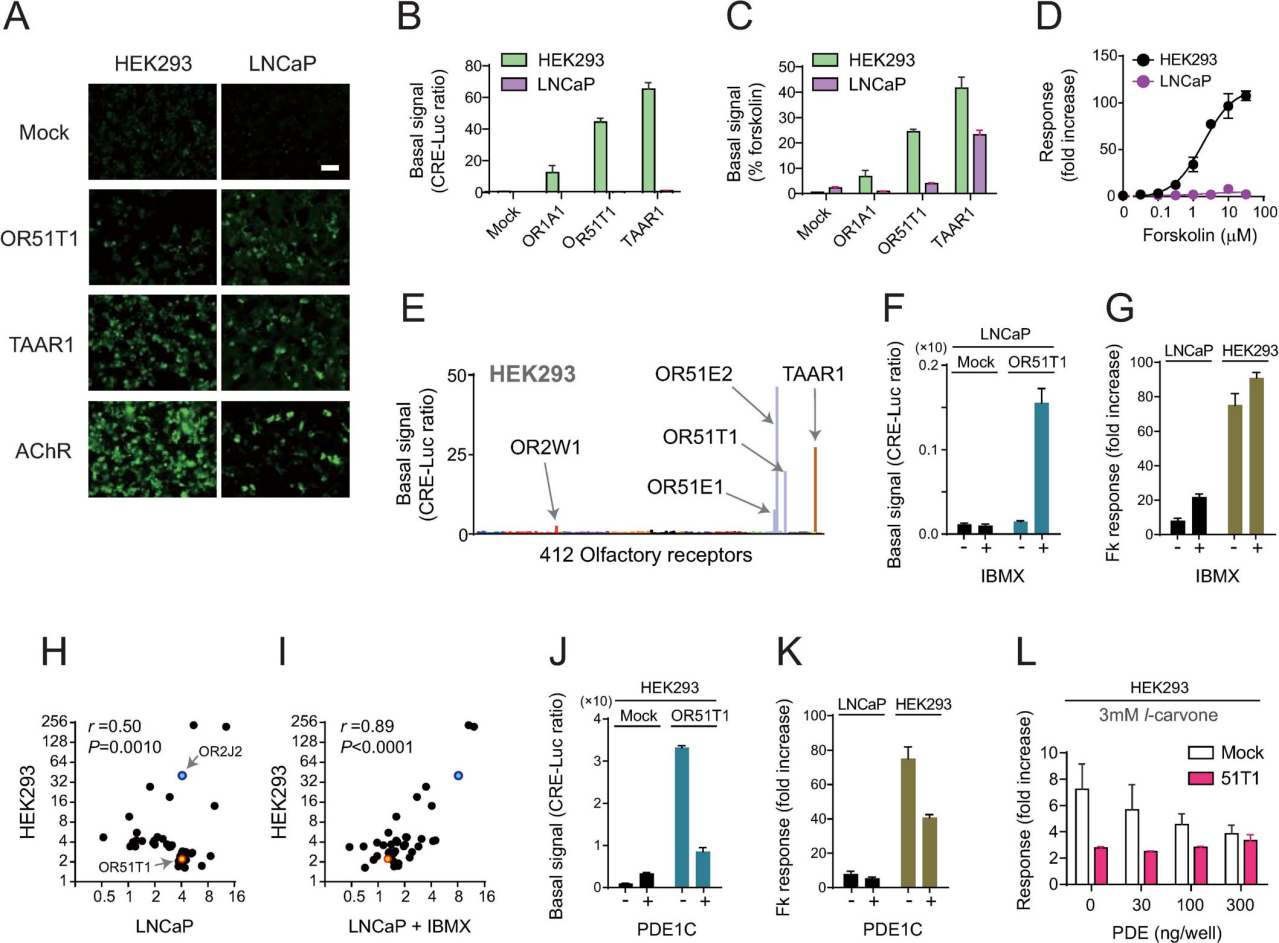
Capacity of HEK293 and LNCaP cells for membrane trafficking of nascent OR proteins and for controlling background intracellular signal from non-stimulated ORs. A. Immunohistochemical analysis (live-cell staining) of HEK293 and LNCaP cells expressing OR51T1 or TAAR1. M3AChR was tested as a positive control of GPCRs for efficient cell-surface expression. All three tested receptors were expressed as fused proteins with an N-terminal FLAG-tag, and they were detected using an anti-FLAG antibody. Scale, 100 μm. B, C. Difference in background signal levels from constitutive activity of ORs in HEK293 (green) and LNCaP (purple) cells. Each type of cell was transfected with CRE/luc2PpGL4.29, pRL-CMV, RTP1S and Gαolf. X-axis indicates transfected ORs. After 24 h of transfection, cells were stimulated with growth medium without odorants, and luciferase activity was measured. Data are presented as CRE-Luc ratio (luminescence intensity of firefly luciferase divided by the luminescence intensity of Renilla luciferase) (B) or %forskolin (C) where CRE-Luc ratio in each transfection condition is presented as normalized values and CRE-Luc ratio in response to 10 μM forskolin was set to 100%. D. Dose-response of HEK293 cells and LNCaP cells to forskolin. Each type of cell was transfected with an empty vector, CRE/luc2PpGL4.29, and pRL-CMV. E. Background signal levels of 412 ORs expressed in HEK293 cells are shown as CRE-Luc ratios. HEK293 cells were transfected with CRE/luc2PpGL4.29, pRL-CMV, and RTP1S. Color bars represent OR families. F. IBMX (0.1 mM) treatment increased OR51T1-mediated basal signal. G. IBMX (0.1 mM) treatment enhanced 10 μM forskolin-mediated responses more robustly in mock-transfected LNCaP cell than in HEK293 cells. H, I. Correlation analysis of response amplitudes of 40 ORs to the odorant mixture in HEK293 cells or LNCaP cells. The 40 ORs were those selected to have response amplitudes above mean + 2SD from all examined ORs in each cell line of the primary screening (Fig 2). X- and Y-axis indicates log2 of response amplitude (fold increase) in each cell line. The Pearson’s correlation coefficient (r) and its p-value are displayed on top of each panel. J. Co-expression of PDE1C (20 ng/well) decreases OR51T1-derived basal signal in HEK293 cells. K. Co-expression of PDE1C decreases 10 μM forskolin-mediated responses drastically in mock-transfected HEK293 cells than in LNCaP cells. L. Co-transfection of different amounts of plasmid coding PDE1C failed to elicit an *l*-carvone-mediated response in OR51T1-expressing cells as compared to that in mock-transfected control. Data are shown as mean ± SE of three to four independent experiments (B-G, and J-L).

Next, to understand the range of applicable ORs and factors that are effective for functional expression in LNCaP cells, we addressed the mechanism by which LNCaP cells allowed the detection of OR51T1 activation. One potential reason was that LNCaP cells efficiently promoted the cell surface expression of OR51T1, but HEK293 cells did not. However, OR51T1 was detected on the surface of HEK293 as well as LNCaP cells in live-cell staining experiments to detect the N-terminal FLAG-tag of OR51T1 proteins (Fig 5A). We also found that the basal signal level from OR51T1 under no ligand stimulation was significantly higher in HEK293 cells than in LNCaP cells (Fig 5B and 5C). This difference in OR51T1-derived basal signal between HEK293 and LNCaP cells seemed to be attributed to the difference in the capacity of cAMP production in each cell because forskolin, a potent adenylate cyclase activator, induced higher cAMP-mediated response in HEK293 cells than in LNCaP cells (Fig 5D). However, the difference in OR51T1-mediated basal signal from each cell line was not proportional to the capacity of cAMP production, and they were still clear when the basal signals (CRE-Luc ratio, Fig 5B) in response to 10 μM forskolin were normalized to 100% (Fig 5C). Thus, HEK293 cells produced large basal signals from a specific repertoire of ORs as reported previously (Fig 5E) [52], and a high basal signal may impede the sensitive detection of ligand-mediated response of OR51T1. A reverse experiment supported this notion. Treatment with 3-isobutyl-1-methylxanthine (IBMX), an inhibitor for phosphodiesterase (PDE), allowed LNCaP cells to produce larger OR51T1-mediated basal signal and enhanced forskolin-mediated response of mock-transfected LNCaP cells more robustly than HEK293 cells (Fig 5F and 5G). Notably, IBMX-treated LNCaP cells reconstituted the HEK293 cell-like pattern of OR activations to the odorant mixture: responses of LNCaP-specific ORs with high basal activity (i.e. OR51T1) were reduced, whereas those of other ORs activated strongly in HEK293 cell were enhanced (i.e. OR2J2, S3 Table). We analyzed the correlation of response amplitudes of ORs to the odorant mixture in HEK293 cells and LNCaP cells with or without IBMX treatment (Fig 5H and 5I). For this analysis, we selected the 40 ORs which showed response amplitudes above mean + 2SD from all examined ORs in each cell line of the primary screening (Fig 2 and S3 Table). The pattern of OR responses in HEK293 cells showed a weak correlation with that in untreated LNCaP cells, but showed a stronger correlation when LNCaP cells were treated with IBMX (*r* = 0.50, *P* = 0.0010 v.s. *r* = 0.89, *P*<0.0001). Thus, we reasoned that depleting the background signal allowed HEK293 cells to detect activation of OR51T1 and tested experimental conditions to manipulate the basal level of signals. We tested the effect of co-expression of PDE1C, which was expressed in the cilia of OSNs. We observed that the co-expression of PDE1C induced decreased levels of OR51T1-mediated basal signal in HEK293 cells, suggesting a reduction in the background noise for detection of an agonist-mediated OR51T1 activation (*P*< 0.0001, student’s *t*-test; Fig 5J). PDE1C expressed in LNCaP cells without OR51T1 did not reduce sensitive detection of stimulant-induced cAMP signal of LNCaP cells (*P* = 0.198, student’s *t*-test; Fig 5K). Under this condition, we tested whether activation of OR51T1 against *l*-carvone was detectable in HEK293 cells. However, no significant response was measured even when different concentrations of PDE1C were tested (Fig 5L). In addition, we also tested the reduction of the amount of transfected plasmid for OR51T1. This approach successfully reduced background signal and led to sensitive detection of ligand-mediated activation of two ORs with high basal activity (OR2W1 and OR51E1), but it was not effective to OR51T1 (S9 Fig). These observations highlight the advantage of LNCaP cells, which are effective for the functional characterization of ORs, especially those with a high basal activity in HEK293 cells.

Thus, we searched for another case in which OR with a high basal activity in HEK293 cells was functionally expressed and successfully assayed in LNCaP cells. Our data on background signals from HEK293 cells transfected with each of the 412 ORs confirmed that OR51T1 elicited a relatively high background signal and identified other ORs with a high basal signal (Fig 5E and S4 Table). These data should be interpreted with caution because the majority of OR species were not recruited on the cell surface of HEK293 cells, and the present data missed a substantial number of ORs with high basal activity. Among the identified receptors with a high basal activity, we were particularly interested in TAAR1, a receptor for organic amine compounds, though the human olfactory epithelium does not express TAAR1 at all or only slightly expresses it [53, 54]. We focused on TAAR1 because TAAR1-expressing LNCaP cells in the initial screening experiment seemed to be responsive to the odorant mixture, although the response amplitude did not meet the criteria described above. The response values showed an average fold increase of 2.8 in TAAR1-expressing LNCaP cells as compared to 1.5 in the vector-transfected control (Fig 2). Thus, the same series of experiments on OR51T1 were conducted using TAAR1. CRE-regulated luciferase reporter gene assays indicated that TAAR1 activation against the mixture and its components was detectable only in LNCaP cells, and it revealed novel types of ligands, *l*-carvone, anis aldehyde, and *cis*-3-hexenol (Figs 3 and 4B). The observed agonistic activity of these odorants was significant but weak because higher concentrations were required for detecting activation of TAAR1 than those of known agonists of organic amines (S10 Fig, [53, 55]). Both HEK293 and LNCaP cells were capable of recruiting TAAR1 protein on the cell surface (Fig 5A), whereas HEK293 cells showed higher background signals when they were expressed with TAAR1 than did LNCaP cells (Fig 5B and 5C). Taken together, our results suggest that the LNCaP cell line is an effective heterologous cell system for functional characterization of a specific repertoire of ORs with high basal activity.

## Discussion

Since the discovery of the OR gene family in 1991 [1], many studies have highlighted the importance of the functions of individual ORs. Genetically manipulated animals lacking a specific OR showed abnormal behavioral olfactory responses in terms of sensitivity, preference, and social communication [39, 56–61]. In humans, taking advantage of the natural knockout of ORs due to genetic polymorphism, a significant function of perception and potential ligands of respective ORs have been suggested [6, 7, 62–65]. However, pairing ORs with cognate ligands has progressed slowly due to their inefficient functional expression in heterologous cells. The significance of the present study is as follows: 1) it provides a novel methodology for functional assay of ORs by utilizing cell lines that endogenously express functional ORs and are effective as heterologous expression systems, and 2) it identifies 31 novel pairs of ORs and their ligands that will potentially contribute to our understanding of how the sense of smell is constructed and technologically controlled in the future.

Prior to investigating the capability of the three cell lines, we used HEK293 cells to perform control experiments to validate the OR and ligand pairs. Previous reports on identification of OR-ligand pairs are useful for evaluating the consistency of our results [3, 4, 38]. In the present study, we consistently detected the activation of OR2W1, OR5P3, and OR8B3 by *l*-carvone, OR1A1 by *d*-carvone, OR2J3 and OR2W1 by *cis*-3-hexenol, and OR1C1 by linalool (black circles in Table 1). The present study successfully identified novel OR-ligand pairs, including OR5K1, OR8H1, and OR10A6 for *l*-carvone; OR5P3 and OR10A6 for *d*-carvone; OR1A1, OR4S2, OR5P3, and OR10A6 for *cis*-3-hexenol; OR1A1, OR2J2, OR2J3, OR5K1, OR5P3, OR8B3, and OR10A6 for anis aldehyde; OR1A1, OR1D2, OR2J2, OR2W1, OR5P3, OR8B3, OR8H1, and OR10A6 for methyl β-naphthyl ketone; and OR1A1 for linalool (white circles in Table 1). However, our primary screening missed OR-ligand pairs identified in previous studies: OR1A1 for *l*-carvone; OR2W1 and OR8B3 for *d*-carvone; OR2J1, OR2J2, and OR14J1 for *cis*-3-hexenol; OR6P1 for anis aldehyde; OR2J3 for methyl β-naphthyl ketone; and OR1N2 for linalool (black triangles in Table 1). The inconsistency may be attributed to differences in the amino acid sequences of OR variants. As genetic variations in human ORs are abundant, a part of ORs used in this study were variants with different amino acids from their reference sequences. Similarly, previous studies tested a series of ORs which included OR variants with different amino acid residues from those in this study, likely providing different assay outcomes to the same odorants. S5 Table shows a comparison with responsiveness of OR variants reported in previous studies. In addition, the inconsistency with previous studies is likely explained by a difference in tested concentrations. A previous study also conducted an OR screen for five odorants tested in this study: *l*-carvone, *d*-carvone, anis aldehyde, *cis*-3-hexenol, and linalool in a 100 μM concentration for primary screening, whereas our current study used higher concentrations [3]. This difference may explain why a large number of ORs were identified in our study. Potential false negative identifications of ORs in our primary screen using 384-well plates may also cause some discrepancy with previous findings. OR14J1 and 6P1 were not included in our plasmid library at the time of primary screening, and therefore, they were not tested. The activated ORs that were missed may be detected in our assay system after testing them at higher concentrations.

The use of LNCaP cells allowed the deorphanization and functional characterization of OR51T1. OR51T1 was found to be sensitive to structurally dissimilar odorants, and therefore, appeared to be classified into broadly tuned ORs [66–68]. The observed high basal activity of OR51T1-expressing cells was also consistent with the reported characteristics of broadly tuned ORs [67]. One potential olfactory function of this type of OR is to act as an intensity analyzer by providing an easy readout of odor concentrations regardless of the identity of odors in the olfactory system [67]. Future identification of a ligand that specifically activates OR51T1 will allow us to pinpoint its cognate perception and examine its potential function.

Next, agonists for TAAR1 were identified from non-amine compounds, such as *l*-carvone, anis aldehyde, and cis-3-hexenol. These three odorants showed an extremely weak affinity to TAAR1 because the response of cells did not reach saturation up to a concentration of 3000 μM; therefore, their EC50 values could not be determined. This is inconsistent with known monoamine agonists, which show a higher affinity and efficacy with EC50 values of 0.1–100 μM (S10 Fig, [55, 62]). The structural basis underlying the sensitive binding to amines was predicted to involve an essential salt bridge between the amino group of agonists and an aspartic acid on the third transmembrane domain, which is highly conserved in many TAARs, including TAAR5 [69]. The observed lower affinities of identified agonists were probably attributed to the lack of a salt bridge in the interaction. However, the present data suggest a statistically significant activation of TAAR1 against non-amine odorants, which was consistent with the binding capacity of a non-amine odorant toward TAAR5 [70]. Recent studies have further suggested that human TAAR1 is expressed in several brain regions but not in the olfactory epithelium; TAAR1 was proposed as a promising therapeutic target for the treatment of schizophrenia, psychosis in Parkinson’s disease, substance abuse, metabolic syndrome, and obesity [53, 71, 72]. Thus, TAAR1 may not be classified as an OR. Taken together, our results suggest that LNCaP cells have the capacity to functionally express a wide range of receptors with high basal activity, including ORs and other GPCRs outside the nose.

We argue that prostate epithelial cells as well as LNCaP cells may possess appropriate signal to noise ratio of cAMP signal for utilizing ORs with high basal activity, including endogenous OR51E1 and OR51E2 as well as exogenous OR51T1 and TAAR1. On the other hand, HEK293 cells appear to be efficient for analyzing ORs with relatively low basal activity. It is worth noting that assay outcomes in each cell line also depend on the potency of available agonists for target ORs. While the detection of OR51T1 activation was specific in LNCaP cells, OR51E1 with high basal activity could also be analyzed in HEK293 cells (S9 Fig). This apparent discrepancy is likely reconciled by the different agonistic potency between nonanoic acid for fatty acid-selective OR51E1 and the identified agonists for broadly tuned OR51T1, which may recognize structurally diverse molecules with weak affinity. Our assumption is that an approach for depleting basal cAMP signal will lead to the optimization of HEK293 cells capable of detecting activation of ORs with a high basal activity. This hypothesis is partially supported by the converse experiment. Up-regulation of basal cAMP signal in LNCaP cells by IBMX treatment provided a similar assay outcome to HEK293 cells. However, our attempts to reduce basal signal in HEK293 cells did not allow the detection of OR51T1 activation potentially because they also reduced the signal from activated ORs. This result highlights the advantage of LNCaP cells for analyzing a specific repertoire of ORs with a high basal activity. Although a previous study and the current study did not detect response of LNCaP cells to agonistic odorants for endogenous OR51E1 and OR51E2 likely due to their low expression levels (S11 Fig, [46]), the potential responsiveness of the endogenous receptors to a subset of odorants may pose a risk in the use of the cell line as a functional assay system for a given exogenous OR. However, a notable advantage of homologous expression systems for characterizing ORs has been proven in this study as well as by a number of previous studies using olfactory sensory neurons [16, 17, 73, 74]. Future studies will establish a more sensitive condition in LNCaP cells via long-term trial and error optimization of the current assay condition using HEK293 cells.

Several heterologous cell systems, other than HEK293 cells, have been reported previously. For example, vertebrate and insect ORs were successfully analyzed in *Xenopus* oocytes using electrophysiological methods, and a variety of common mammalian cell lines have also been utilized for the successful characterization of ORs [20, 40, 42, 44, 75]. These attempts demonstrated the high consistency of the observed functionality of ORs, regardless of the type of heterologous cells and assay methodologies. However, the limited number of tested ORs failed to provide an understanding of the range of applicable ORs in each type of heterologous cell. The current report tested the highest number of ORs for comparison of multiple cell lines, and it identified cases of cell line-dependent detection of OR activation. Although we have focused only on the LNCaP cell line and ORs with a high constitutive activity, the present data also include potential ORs functionally expressed only in a distinct cell line (S3 Fig). Further investigation using a different set of odorants against ORs expressed in the three cell lines tested here and others will increase the usefulness of cell lines with endogenous ORs as an additional heterologous cell system. Accumulating information on odorant-OR pairs will provide an understanding of how the brain computes odor inputs, constructs the sense of smell, and develops an approach to efficiently control olfactory sense.

## Supporting information

S1 Fig Optimization of transfection conditions for the three cell lines. A-C. Six patterns of transfection conditions, including (i) 0.41 μL of PEI-MAX (condition for HEK293 cells), (ii) 0.2 μL of Lipofectamine 2000, (iii) 0.35 μL of Lipofectamine 2000, (iv) 0.5 μL of Lipofectamine 2000, (v) 0.15 μL of Lipofectamine 3000, and (vi) 0.3 μL of Lipofectamine 3000, were tested. DMEM was used as a transfection buffer for HEK293 cells, and Opti-MEM was used as a transfection buffer for the other three cell lines. Based on the result, the condition adopted for each cell line is highlighted in red. Data are shown as mean ± SE of three independent experiments. D. Buffers for transfection reagent and plasmid for HepG2, HuH7, and LNCaP cells. This figure shows the fold increase values of OR1A1-expressing cells stimulated by l-carvone. Each cell line was tested with Opti-MEM or a growth medium for each cell (DMEM for HepG2 and HuH7 cells and RPMI1640 for LNCaP cells). These data show the average of duplicated wells from one experiment. Based on the result of this experiment, DMEM was used for HEK293 and HuH7, and Opti-MEM was used for HepG2 and LNCaP (Fig 2).
(EPS)

S2 Fig Dose-response analysis of candidate ORs for each odorant (related to Fig 1). HEK293 cells were transfected with each ORs, as well as CRE/luc2PpGL4.29, pRL-CMV, and RTP1S and stimulated with odorants. The response of cells expressing each OR is presented as fold increase. Data are shown as mean ± SE of three independent experiments.
(EPS)

S3 Fig Dose-response of OR-expressing HEK293 cells to the odorant mixture. HEK293 cells were transfected with each OR, CRE/luc2PpGL4.29, pRL-CMV, Gαolf, and RTP1S and stimulated with multiple concentrations of the odorant mixture. X-axis indicates concentration of the odorant mixture. For an example, 1000 μM of odorant mixture was composed of *l*-carvone (100 μM), *cis*-3-hexenol (100 μM), anis aldehyde (100 μM), methyl β-naphthyl ketone (100 μM), *d*-carvone (300 μM), and linalool (300 μM). Data are shown as mean ± SE of three or six replicates from one or two independent experiments. *At least one concentration of the odorant produced a statistically significant response of an OR-expressing cell as compared to both that of cells stimulated with medium alone and a vector-transfected control cell (Sidak-–Bonferroni method with alpha = 5.0%).
(EPS)

S4 Fig Dose-response of OR-expressing HepG2 cells to the odorant mixture. HepG2 cells were transfected with the indicated ORs, CRE/luc2PpGL4.29, pRL-CMV, Gαolf, and RTP1S and stimulated with multiple concentrations of the odorant mixture. Error bars, S.E. from three replicates. *At least one concentration of the odorant produced a statistically significant response of an OR-expressing cell as compared to both that of cells stimulated with medium alone and a vector-transfected control cell (Sidak-–Bonferroni method with alpha = 5.0%).
(EPS)

S5 Fig Dose-response of OR-expressing HuH7 cells to the odorant mixture. HuH7 cells were transfected with the indicated ORs, CRE/luc2PpGL4.29, pRL-CMV, Gαolf, and RTP1S and stimulated with multiple concentrations of the odorant mixture. Error bars, S.E. over three or six replicates from one or two independent experiments. *At least one concentration of the odorant produced a statistically significant response of an OR-expressing cell as compared to both that of cells stimulated with medium alone and a vector-transfected control cell (Sidak-–Bonferroni method with alpha = 5.0%).
(EPS)

S6 Fig Dose-response of OR-expressing LNCaP cells to the odorant mixture. LNCaP cells were transfected with the indicated ORs, CRE/luc2PpGL4.29, PRL-CMV, Gαolf, and RTP1S and stimulated with multiple concentrations of the odorant mixture. Error bars, S.E. over three replicates. *At least one concentration of the odorant produced a statistically significant response of an OR-expressing cell as compared to both that of cells stimulated with medium alone and a vector-transfected control cell (Sidak-–Bonferroni method with alpha = 5.0%).
(EPS)

S7 Fig The effect of co-expression of Gαolf on the assay sensitivity of OR-expressing LNCaP cells. LNCaP cells were transfected with the indicated ORs, CRE/luc2PpGL4.29, and pRL-CMV RTP1S with or without Gαolf. They were then stimulated with multiple concentrations of the odorant mixture. Data are shown as mean ± SE of three independent experiments.
(EPS)

S8 Fig No responsiveness of OR51T1-expressing LNCaP cells to odorants. LNCaP cells were transfected with each OR, CRE/luc2PpGL4.29, pRL-CMV, Gαolf, and RTP1S and stimulated with the indicated concentration of ambrettolide, cedramber, and galaxolide.
(EPS)

S9 Fig Experimental conditions to reduce the basal level of signals failed to detect OR51T1 activation by l-carvone in HEK293 cells. A-C. Reduction of the amount of transfected plasmid improved response of HEK293 cells expressing OR2W1 or OR51E1; examples of previously characterized ORs with a high basal activity that did not allow the detection of OR51T1 activation. HEK293 cells were transfected with each OR, CRE/luc2PpGL4.29, pRL-CMV, and RTP1S.
(EPS)

S10 Fig Response of TAAR1-expressing LNCaP cells and HEK293 cells to known ligands. LNCaP cells and HEK293 cells were transfected with TAAR1, CRE/luc2PpGL4.29, pRL-CMV, Gαolf and RTP1S and stimulated with the indicated concentration of amines. Data are shown as mean ± SE of three independent experiments.
(EPS)

S11 Fig No responsiveness of LNCaP cells to agonists for endogenous OR51E1 and OR51E2. Mock-transfected LNCaP cells were stimulated with the indicated concentration of odorants. LNCaP cells were transfected with CRE/luc2PpGL4.29, pRL-CMV, Gαolf, and RTP1S. Error bars, S.E. from three replicates.
(EPS)

S1 Table OR sequences used in this study. (XLSX)

S2 Table OR screening using HEK293 cells against six odorants (Fig 1). (XLSX)

S3 Table OR screening using four cell lines against an odorant mixture (Fig 2). (XLSX)

S4 Table Background basal activity of 412 ORs expressed in HEK293 cells (related to Fig 5E). (XLSX)

S5 Table Comparison with genetic variants used in previous studies. (XLSX)
